# Care Transitions and Mortality among Skilled Nursing Facility Residents with Opioid Use Disorder

**DOI:** 10.1093/geroni/igaf122.1012

**Published:** 2025-12-31

**Authors:** Patience Dow, Miriam George, Andrew Zullo, Ashley Ritter, Momotazur Rahman

**Affiliations:** Brown University School of Public Health, Providence, Rhode Island, United States; Brown University, Providence, Rhode Island, United States; Brown University, Providence, Rhode Island, United States; Hunter-Bellevue School of Nursing, Hunter College, City University of New York, Yardley, Pennsylvania, United States; Brown University, Providence, Rhode Island, United States

## Abstract

There is growing demand for skilled nursing facility (SNF) services among individuals with opioid use disorder (OUD) for reasons including rising rates of chronic medical conditions and serious infections. Little is known about the characteristics and outcomes of people with OUD admitted to SNFs. We examined differences in health service use and mortality between SNF residents with and without OUD. Using 2016-2020 100% Medicare inpatient claims, we identified hospitalizations with discharge to SNFs. We matched each beneficiary with OUD with up to 5 without OUD based on age, sex, low-income subsidy status, and residential county. Outcomes were hospital readmissions, home health service use, and all-cause mortality within 180 days following hospital discharge. Inverse-probability weighting (IPW) adjusted for demographics, state, year of SNF admission, comorbidity burden, hospital length of stay, intensive care, and select principal diagnoses. Unadjusted and IPW-adjusted risk differences (RDs) were calculated. Among 30,922 beneficiaries with OUD matched to 137,454 without OUD (mean age: 71 years), those with OUD had higher unadjusted risks for readmissions (44.5% vs. 27.9%) and home health use (43.2% vs. 35.4%) but lower mortality risk (16.5% vs. 17.8%). After IPW adjustment, beneficiaries with OUD remained at higher risk for readmissions (RD = 15.8%; 95% CI:15.1%,16.5%) and home health use (RD = 9.6%; 95% CI:9.1%,10.2%) but had no difference in mortality risk (RD=-0.4%; 95% CI:-0.8%,0.1%). Best practices for providing care to individuals with OUD and co-occurring skilled nursing needs must be a near-term focus for post-acute and long-term care practice and policy to help reduce concerning differences in readmission rates.

